# Unexpected Association of Desacyl-Ghrelin with Physical Activity and Chronic Food Restriction: A Translational Study on Anorexia Nervosa

**DOI:** 10.3390/jcm9092782

**Published:** 2020-08-28

**Authors:** Philibert Duriez, Lauralee Robichon, Roland Dardennes, Guillaume Lavoisy, Dominique Grouselle, Jacques Epelbaum, Nicolas Ramoz, Philip Gorwood, Virginie Tolle, Odile Viltart

**Affiliations:** 1 Institute of Psychiatry and Neuroscience of Paris (IPNP), Université de Paris, INSERM UMR-S 1266, F-75014 Paris, France; p.duriez@ghu-paris.fr (P.D.); lauralee.robichon@sfr.fr (L.R.); dominique.grouselle@inserm.fr (D.G.); jacques.epelbaum@inserm.fr (J.E.); nicolas.ramoz@inserm.fr (N.R.); p.gorwood@ghu-paris.fr (P.G.); virginie.tolle@inserm.fr (V.T.); 2 GHU Paris Psychiatrie et Neurosciences, Hôpital Sainte-Anne, F-75014 Paris, France; r.dardennes@ghu-paris.fr (R.D.); guillaumelavoisy@hotmail.com (G.L.); 3 UMR 7179 CNRS, MNHN, Adaptive mechanism and Evolution (MECADEV), 91800 Brunoy, France; 4 Cité scientifique, SN4, Université de Lille, 59491 Villeneuve d’Ascq, France

**Keywords:** restrictive anorexia nervosa, weight recovery, animal models, acyl-ghrelin, desacyl-ghrelin, physical activity, chronic food restriction

## Abstract

Anorexia nervosa (AN) is a severe metabopsychiatric disorder characterised by caloric intake restriction and often excessive physical exercise. Our aim is to assess in female AN patients and in a rodent model, the co-evolution of physical activity and potential dysregulation of acyl—(AG) and desacyl—(DAG) ghrelin plasma concentrations during denutrition and weight recovery. AN inpatients were evaluated at inclusion (T0, *n* = 29), half—(T1) and total (T2) weight recovery, and one month after discharge (T3, *n* = 13). C57/Bl6 mice with access to a running wheel, were fed ad libitum or submitted to short—(15 days) or long—(50 days) term quantitative food restriction, followed by refeeding (20 days). In AN patients, AG and DAG rapidly decreased during weight recovery (T0 to T2), AG increased significantly one-month post discharge (T3), but only DAG plasma concentrations at T3 correlated negatively with BMI and positively with physical activity. In mice, AG and DAG both increased during short- and long-term food restriction. After 20 days of ad libitum feeding, DAG was associated to persistence of exercise alteration. The positive association of DAG with physical activity during caloric restriction and after weight recovery questions its role in the adaptation mechanisms to energy deprivation that need to be considered in recovery process in AN.

## 1. Introduction

Anorexia nervosa (AN) is a psychiatric disorder where the severe weight loss due to a reduction on food intake is associated with high levels of physical activity. Indeed, 31% to 80% of AN patients display inappropriate quantity of exercise with respect to their energy resources [1,2]. Hyperactivity has been associated with an increase in the length of hospitalisation stay [3], a poor treatment outcome both interfering with refeeding therapies and increasing the risk of relapse [4]. One out of two patients relapses within a year following inpatient treatment and approximately 20% of patients experience recurrent patterns of remission and relapse or chronic disease [5,6]. The disorder affects predominantly women and girls, with female to male ratios of approximately 10/1 to 15/1 [7]. The etiology of AN is complex, but recent evidences emphasise metabolic and endocrine aspects as key pathophysiological determinants of the disorder [8,9,10,11,12].

Amongst many metabolic and endocrine factors, ghrelin a 28-amino-acid peptide orexigenic gut hormone produced by the X/A-like endocrine cells in the oxyntic glands of the gastric fundus [13,14,15], is involved in many physiological processes associated with feeding and exercise including the regulation of energy metabolism and appetite [16], the cardiovascular system as autonomic nervous system [17,18,19,20], and the modulation of reward and motivation [11,21,22]. Ghrelin has rapidly been considered as a biomarker of AN since its plasma concentrations are significantly increased in AN patients and return to control values after renutrition as also observed in animal models mimicking several symptoms of AN [23,24,25]. Acylation (on its third serine residue) by ghrelin O-acyl transferase allows acyl-ghrelin (AG) to bind to its receptor, the growth hormone secretagogue receptor (GHSR) type 1a that is widely distributed within the central nervous system [13,26]. Desacyl-ghrelin (DAG) is also present in the blood circulation [27]. Original studies using competition assays showed that DAG accounts for 80–90% of total circulating ghrelin [28]. More recently, using very selective sandwich immunoassays for AG and DAG, DAG accounted for 76% of the total circulating ghrelin (i.e., AG+DAG) [29]. Despite a number of studies reporting a physiological role for DAG, usually opposite to AG [27,30,31,32], its role remains elusive.

Beside appetite, many clinical and preclinical studies support an association of ghrelin with physical exercise. More particularly, in healthy subjects, acute or short-term exercise duration is associated with a decrease in ghrelin plasma concentrations while chronic physical activity is correlated with increased concentrations of ghrelin [33,34,35]. Increased ghrelin plasma concentrations are also described in rodents performing chronic treadmill exercise [36]. In pathological situations associated with excessive physical activity such as AN, total ghrelin is positively correlated with physical activity, measured by daily step counts [37]. In the activity-based anorexia (ABA) model, where time-restriction in food access is associated with running activity, mice display excessive daytime physical activity in the context of limited access to food [38,39]. Interestingly, in ABA mice, a single intracerebroventricular injection or chronic peripheral treatment with a GHS-R1a antagonist lead to a significant decrease of daytime activity [38]. Moreover, GHS-R KO mice show a more rapid exhaustion in an endurance exercise as compared to wild-type mice [40]. However, the differential effect or association of AG and DAG with chronic physical activity, in clinical or preclinical studies is controversial, because of the kind of exercise considered (acute, endurance, etc. …) or the sex of the participants (usually males, in human or rodents). Furthermore, in healthy conditions, only plasma AG concentrations were modulated by exercise [41,42,43]. Finally, most of the studies that investigated the role of ghrelin in AN have focused on variations in plasma total ghrelin or AG, neglecting the potential involvement of DAG. To our knowledge, longitudinal data are not yet available on AG and DAG levels during a refeeding hospital program and after body weight recovery associated with physical activity. A standardised definition of remission, recovery and relapse is still lacking [44]. We thus hypothesised that in long-term food restriction and in nutritional recovery, AG and DAG plasma concentrations evolve differentially according to the level of physical activity. For this purpose, we first assessed the evolution of plasma concentrations of AG and DAG in ill- and recovered-AN patients, in relation with their physical activity. Then, we used a modified mouse ABA model of chronic quantitative food restriction associated with a voluntary running activity in a wheel, to determine whether AG and DAG plasma concentrations reflect the level of physical activity during the periods of chronic food restriction and nutritional recovery [45,46]. Here, “nutritional recovery” is used to identify patients or rodents that followed a program of refeeding and included weight restoration.

## 2. Materials and Methods

### 2.1. Experiment 1. Clinical Investigation

*Participants*. Twenty-nine female patients suffering from anorexia nervosa (AN) in undernourished state were included in the study (Table 1). Thirteen patients had full weight recovery during intensive in-patient program and were evaluated one-month post discharge. Participants, aged 18–37 years, fulfilled DSM-V criteria for AN (APA, 2013). All patients attended a structured in-patient program in the Eating Disorders Unit of *Clinique des Maladies Mentales et de l’Encéphale* (CMME, Sainte-Anne Hospital, Paris, France) and received behavioural nutritional rehabilitation program with progressive and controlled access to physical activity and were discharged after reaching their target weight and maintaining it for at least 2 weeks. We excluded patients with active malignancies, active inflammatory or infectious diseases, epilepsy, and other psychiatric disorders. Several clinical parameters were recorded at the different time-points. Patients were weighted at every inpatient and follow-up assessment, size was measured and corresponding body mass indexes (BMIs) were calculated. Physical activity was assessed with the self-reported International Physical Activity Questionnaire (IPAQ) [47]. The study protocol was approved by *Comité de Protection des Personnes Ile de France III* (EUDRACT N: 2008-A008 17–48; CPP N Am5355-2-2592). All patients gave written informed consent prior to participation. All data were recorded anonymously.

In order to assay hormonal longitudinal variations in different nutritional conditions, blood samplings were performed in undernourished conditions (T0) (5–8 days after the arrival at the Hospital) during the acute phase of the disorder, in the course of the refeeding process (T1: 50–70% target BMI of 20 kg/m^2^), after complete weight recovery (T2: 90–100% target BMI) or during the stabilisation process (T3: 1 month following discharge).

*Conditions of Blood Sampling, Processing and Storage of Blood*. Blood sampling was performed after an overnight fast. Blood was collected on tubes containing 15% EDTA and Aprotinin 250 KIU (tube BD vacutainer EDTA K3 Aprotinin). Blood samples were immediately centrifuged at 4 °C (1000× *g* for 15 min). In addition, plasma samples were aliquoted and supplemented with HCl at a final concentration of 0.1 N immediately after collection in order to preserve acylation and frozen at −80 °C.

*Acyl—and Desacyl-Ghrelin Immunoassays*. Acyl- (AG) and desacyl-ghrelin (DAG) concentrations were assayed in duplicates with selective two-sites sandwich enzyme-immunoassays (EIA) using two different monoclonal antibodies for capture and revelation (human AG and DAG EIA Easy Sampling Elisa kits, Ref A05306 and Ref A05319, respectively, Bertin Bioreagent, Montigny-le-Bretonneaux, France). For AG, the limit of detection is 4 pg/mL. Intra- and inter-assay coefficients of variation are 9 and 16%, respectively. For DAG, the limit of detection is 10 pg/mL. Intra- and inter-assay coefficients of variation are 6 and 16%, respectively.

### 2.2. Experiment 2. Preclinical Investigation

*Animals*. Seven-week old C57BL/6J female mice (Charles River Laboratories, L′Arbresle, France) weighing 18.3 ± 0.1 g were housed by cages of two to avoid isolation stress and hypothermia [45]. They were kept in a pathogen-free barrier facility maintained at 22–24 °C with a 12:12-h dark-light cycle (lights on at 07:00 a.m.). During one week of habituation, mice were weighted every day to get acclimatised to handling. Mice within the same cage had a similar initial body weight. They had free access to water and to standard chow diet (3% fat, 16% protein, 60% carbohydrate, 4% fibres, 2.79 kcal/g; Safe A04). All experiments were carried out in accordance with the European Communities Council Directive (86/609/EEC) and approved by the Regional ethical committed of Paris Descartes University France.

*Short-Term Food Restriction Protocol*. In this first set of experiments, mice were randomised into two experimental groups according to their initial body weight. Mice of the group “ad libitum and wheel” (group ALW, *n* = 12) were placed in a cage equipped with a free running wheel (ActiviWheel Software; Intellibio, Seichamps, France) and had free access to food. In the group “food restriction and wheel” (group FRW, *n* = 12), mice were placed in a cage equipped with a free running wheel and exposed to a 30% quantitative food restriction for three days followed by 50% quantitative food restriction through the end of the protocol. This restriction was calculated every day from the total food consumed by each mouse in the ALW group the previous day, by weighing the whole pellets in the feeder. Food (on pellet per mouse) was distributed directly into the cage every day at 6:30 p.m. Body weight was monitored daily at the same time. In 8 out of the 12 cages, the locomotor activity was assessed daily with a running wheel (diameter: 230 mm; width: 50 mm; 1 revolution = 0.72 m) linked to a computer system that measured interval counts (10 min) per mean wheel revolution (ActiviWheel Software; Intellibio, Seichamps, France). In 4 out of the 6 cages of ALW mice, the physical activity was measured with a manual counter (Sigma Germany BC 9.16 ATS), which indicated total distance, mean speed, and maximum speed in 12-h periods. Manual counters were read twice per day: between 7:30 and 8:00 a.m. to evaluate ALW nocturnal activity and around 6:00 p.m., before the food distribution, for the diurnal activity. The wheel running activity was measured per cage, because ethologically speaking and for the welfare of the animals, we decided to avoid stress isolation and the hypothermia induced by our long-term caloric restriction protocol. The recording of physical activity thus reflected the activity of two mice that usually run two by two in the wheel (personal observation), resulting in 6 ALW and 6 FRW cages. Since we measured each animal body weight daily, we were able to detect changes in feeding or running activity and thus avoided the cage-effect of these analyses. As mentioned, weight loss in a same cage is usually similar (considering the standard deviation) leading us to consider the cage value of physical activity in wheel for each mouse. Data were extracted with an excel macro (Microsoft Office Standard, 2016) to obtain cumulative activity or day/night activity. To establish a link between plasma concentrations of AG and DAG with physical activity, blood samples were collected twice: in the morning (D11) at 9:00 a.m. and before food distribution (at D10) at 5:00 p.m., when the anticipatory food activity was developed in the FRW mice (see results).

*Long-Term Food Restriction and Nutritional Recovery Protocols*. In the second set of experiments, mice were randomised according to their initial body weight into two experimental groups FRW (*n* = 6) and ALW (*n* = 6) as described above. The experimental protocol was exactly similar to the short-term food restriction, except that we maintained the groups of mice in this protocol for 8 weeks. Body weight was monitored three times a week. The locomotor activity was assessed daily with a running wheel linked to a computer system that measured interval counts (10 min) per mean wheel revolution (ActiviWheel Software; Intellibio, Seichamps, France). After 8 weeks of this protocol, nutritional recovery was achieved by giving ad libitum access to standard diet to all FRW mice while free access to the running wheel was maintained. As mentioned above, the activity was evaluated per cage. Data were extracted with an Excel^®^ macro (Microsoft Office Standard, 2016) to obtain cumulative activity or day/night activity. To evaluate the kinetic of plasma ghrelin concentrations during the refeeding period, two blood samples were withdrawn at 5:00 p.m.: on day 51, one day after the beginning of the refeeding, and on day 70, at sacrifice (after two weeks of nutritional recovery).

*Blood Samples Collection. AG and DAG Plasma Assays*. Blood samples were collected from the caudal vein with a 1-mL syringe in EDTA coated tubes (1 mg/mL final, Microvette^®^ CB 300 μL, Sarstedt, Germany) containing p-hydroxy-mercuribenzoic acid (PHMB 0.4 mM final), which is a serine protease inhibitor, and kept at 4 °C until processing. At the end of the short- and long-term protocols, mice were deeply anesthetised with an overdose of ketamine (100 mg/kg) and xylazine (20 mg/kg) mix. Blood was collected through cardiac puncture with a 1-mL syringue and transferred into EDTA coated tubes. Samples were rapidly centrifuged (1000× *g* for 10 min, 4 °C), to collect plasma (around 80 μL), which was immediately acidified with HCl (0.1 N final) to preserve ghrelin acylation. Plasma aliquots were frozen in dry ice before being stored at −80 °C until they were assayed. Plasma AG and DAG concentrations were evaluated by specific EIA (A05118 for the acylated form and A05117 for the des-acylated form; Bertin Bioreagents, Montigny le Bretonneux, France). All samples were analysed in duplicates. Intra- and inter-assay coefficients of variations were 6.1% and 5.7% for AG and 5.5% and 4.8% for DAG, respectively.

*Statistical Analysis*. Analysis of normality and equality of variances were tested by Shapiro-Wilk test. Statistical analysis were performed using one-way ANOVA followed by a Fisher *post-hoc* test when the *p* value of the ANOVA was significant (*p* < 0.05) or a non-parametric ANOVA followed by Tukey or Bonferroni post hoc test was used when appropriate, using Statview^®^ software (SAS institute Inc., Cary, NC, USA).

Clinical values are given as mean ±SD. Preclinical values are given as mean ±SEM. For both clinical and preclinical values correlations we used Pearson correlation test if normality is respected (Shapiro-Wilk *p* > 0.05) or Spearman’s rank correlation coefficient if not (Shapiro-Wilk *p* < 0.05). We used Jamovi Softare (Version 1.1; R Core Team 2018). The level for significance was established at 5%. We considered a tendency at 0.1 > *p* > 0.05. Graphs were generated using GraphPad Prism^®^ 5.01 (Abacus Concepts, Berkeley, CA, USA).

## 3. Results

### 3.1. Experiment 1. Clinical Investigation

#### 3.1.1. Longitudinal Evolution of BMI and Physical Activity during Inpatient Weight Recovery and One-Month Post Discharge

At inclusion (T0), 29 female patients were evaluated. Only one patient left the protocol before T1. Thirteen patients out of the 29 were re-evaluated both at T2 (90–100% target BMI) and one-month post-discharge (T3). Our analysis focused on recovery and early modifications of metabolic parameters after discharge (Time T3). We thus considered only patients who succeeded in obtaining “full” weight recovery during treatment in our clinical unit, namely the 13 patients presented in our study. The other patients (*n* = 16) did not fulfil the criteria and left hospital mainly after “partial” weight recovery (about 16 kg/m^2^). Table 1 describes the clinical sample.

**Figure 1:**
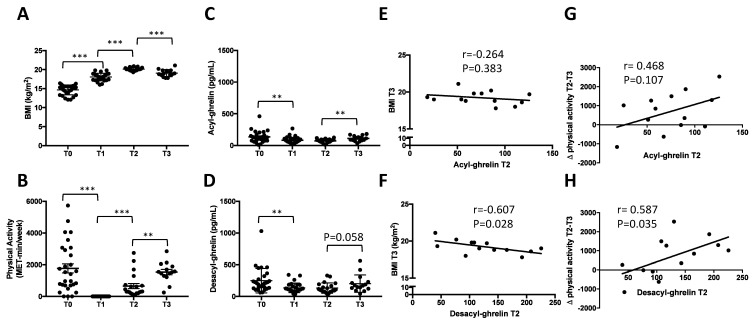
Longitudinal evolution of acyl-ghrelin (AG) and desacyl-ghrelin (DAG) during inpatient weight restoration and one-month post discharge. One-month post hospitalisation (T3), BMI (**A**) and physical activity (**B**) significantly decreased and plasma concentrations of AG (**C**) increased but not DAG (**D**). Plasma concentrations of DAG at the end of hospitalisation (T2) were negatively correlated to BMI one month later (T3,**F**), but not AG (**E**) and positively correlated to the increase of physical activity between the end of hospitalisation and one month later (**H**), but not AG (**G**). Only statistical differences between two consecutive time-points are reported. T0 = 1-week post-admission; T1 = 50–70% of target BMI reached; T2 = discharge BMI (close to target BMI); T3 = 1-month post-discharge visit. BMI: body mass index. MET: metabolic equivalent of task. ** *p* < 0.01; *** *p* < 0.001.

Between T0 and T2, patients increased their BMI according to the strict clinical protocol with weight therapeutic contract (*U* = 51, *p* < 0.001) and reduced their physical activity (*U* = 53.5, *p* = 0.002). The BMI significantly increased between T0 and T2 (F_(2-75)_ = 186, *p* < 0.001; Figure 1A). More specifically, post-hoc analysis revealed a significant increase between T0 and T1 (*p* < 0.001) and between T1 and T2 (*p* < 0.001). Physical activity was permitted after 50% of total expected weight gain, explaining no physical activity reported at T1 (Figure 1B).

#### 3.1.2. Rapid Decrease of Circulating AG and DAG during Refeeding Period

Both AG and DAG plasma concentrations significantly decreased during weight recovery (respectively F_(2-75)_ = 7.68, *p* < 0.001; F_(2-75)_ = 6.86, *p* = 0.002, Figure 1C,D). More specifically, post-hoc analysis revealed a significant decrease of these two forms of ghrelin between T0 and T1 (AG: *p* = 0.006; DAG: *p* = 0.005), T0 and T2 (AG: *p* = 0.003; DAG: *p* = 0.009) and T1 and T2 (AG: n.s.; DAG: n.s.). The AG/DAG ratio was calculated, and ANOVA analysis did not show any significant effect of time (F_(2-75)_ = 0.04, *p* = 0.963).

#### 3.1.3. Early Increase of Circulating AG but Not DAG One Month after Discharge

One month after discharge (T3), patients BMI globally decreased (20.1 ± 0.09 vs. 19.1 ± 0.24; T2 vs. T3, *U* = 51, *p* < 0.001, Figure 1A). Physical activity was significantly increased (T2 vs. T3, *U* = 53.5, *p* = 0.002, Figure 1B). Plasma concentrations of AG were significantly increased (73.2 ± 30.9 vs. 111 ± 43.6; T2 vs. T3, *U* = 79, *p* = 0.011, Figure 1C), with mean circulating DAG at T3 was 153% of that at T2 (*U* = 08, *p* = 0.058, Figure 1D). The AG/DAG ratio was calculated and no significant difference was noted between T2 vs. T3 (*U* = 146, *p* = 0.727).

#### 3.1.4. Correlations between Circulating DAG after Weight Recovery (T2) and BMI at T3

The correlations are presented in Table 2. At T2, AG and DAG plasma concentrations were not correlated to BMI (respectively: r = 0.107, *p* = 0.73; r = −0.378, *p* = 0.202). DAG plasma concentrations, but not AG, were positively correlated with physical activity (respectively: rho = −0.609, *p* = 0.027; rho = -0.322, *p* = 0.283). Furthermore, DAG value at T2 was negatively correlated with BMI at T3 (r = −0.607, *p* = 0.028, Figure 1F), while it was not the case for the AG value (r = −0.264, *p* = 0.383, Figure 1E). Finally, the increase of physical activity between T2 and T3 was significantly correlated to DAG value at T2 (r = 0.587, *p* = 0.035 Figure 1H), but not AG value at T2 (r = 0.468, *p* = 0.107, Figure 1G).

### 3.2. Experiment 2. Preclinical Investigation

#### 3.2.1. Short Term Food Restriction Protocol: Link between AG, DAG and Physical Activity

**Figure 2:**
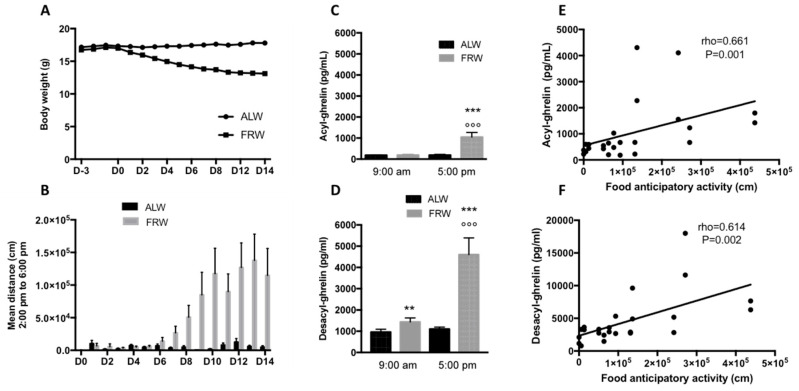
Short-term food restriction protocol. (**A**) Body weight evolution with a significant decrease from D2 to D14 for FRW vs. ALW mice. (**B**) Implementation of the food anticipatory activity (FAA, day activity) in the FRW mice from D6 to D14. (**C**) Mean plasma concentrations of acyl- (AG) and (**D**) desacyl-ghrelin (DAG) sampled at D10 (5:00 p.m.) and D11 (9:00 a.m.). Significant increases of AG and DAG were observed between morning and late afternoon samples only for FRW mice. (**E**,**F**) Plasma concentrations of AG (**E**) and DAG (**F**) were positively correlated with food anticipatory activity. Data are expressed as mean ± SEM; *n* = 24/group. °°° *p* < 0.001 9 h vs. 17 h; ** *p* < 0.01, *** *p* < 0.001 ALW vs. FRW. ALW: ad libitum and wheel; FRW: food restriction and wheel.

At D0, the body weight was not significantly different between ALW and FRW mice (17.3 ± 0.25 vs. 17.0 ± 0.19 g). At D14, the body weight of FRW mice was significantly decreased (12.92 ± 0.21 g) compared to ALW mice (17.89 ± 0.27; t = 14.63, *p* < 0.0001; Figure 2A). Physical activity was evaluated per cage (2 mice per cage). Total 24 h activity was similar between ALW and FRW at D0 (*U* = 71, *p* = 0.96; ALW vs. FRW: 564,302 ± 196,102 vs. 348,602 ± 113,549) and at D14 (*U* = 50, *p* = 0.22; ALW vs. FRW: 1,032,292 ± 308,989 vs. 564,872 ± 183,051). From D8, only FRW mice developed a food anticipatory activity (FAA, *p* < 0.01 FRW vs. ALW; Figure 2B) 4 h 30 before the distribution of food (2:00 *p*.m. to 6:30 p.m.). From D10 to D14, ANOVA analysis for repeated measures revealed an interaction between group and day/night activity (F_(1-160)_ = 8.86, *p* = 0.049). When considering the cumulative activity between D10 and D14, post-hoc analysis indicated that ALW showed the highest activity during the night (day vs. night: 52,178 ± 18,874 cm vs. 4,850,005 ± 1,358,516 cm, *p* = 0.008), and FRW mice did not show any significant differences between day and night activity (day vs. night: 877,885 ± 189,871 cm vs. 1,026,869 ± 189,585 cm, *p* = 0.61) reflecting the FAA.

Plasma ghrelin concentrations were measured when the FAA was clearly developed: before distribution of food for FRW (at 5:00 p.m.) and when mice were fed (at 9:00 a.m.). At 9:00 a.m. AG plasma concentrations in fed mice were not different between ALW and FRW (*p* = 0.15), but were significantly increased only for FRW mice at 5:00 p.m. as compared to ALW mice (*U* = 79.5, *p* < 0.0001, Figure 2C). DAG plasma concentrations were significantly higher in FRW mice both at 9:00 a.m. (*U* = 151, *p* = 0.0041) and at 5:00 *p*.m. (*U* = 32, *p* < 0.0001) than in ALW mice (Figure 2D). Finally, AG and DAG were significantly increased between morning and afternoon (*p* < 0.0001, Figure 2C,D). The AG/DAG ratio was significantly different between ALW and FRW at 9:00 a.m. (0.22 ± 0.02 vs. 0.13 ± 0.01, *U* = 140, *p* = 0.0018), but not at 5:00 p.m. (0.25 ± 0.04 vs. 0.24 ± 0.03, *U* = 250.5, *p* = 0.44).

The high quantity of physical activity performed by FRW mice during FAA (day 12) was correlated with high plasma concentrations of AG (Figure 2E, rho = 0.661, *p* = 0.001) and DAG (Figure 2F; rho = 0.614, *p* = 0.002).

#### 3.2.2. Long Term Food Restriction Protocol and Refeeding

**Figure 3:**
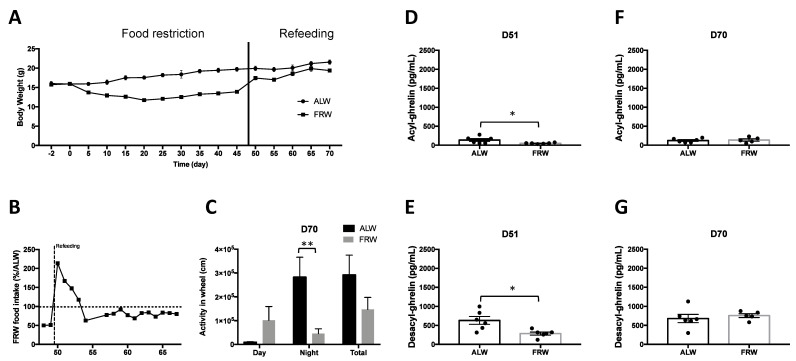
Long-term food restriction protocol and nutritional recovery (refeeding). (**A**) Longitudinal evolution of body weight during long term protocol. (**B**) Evolution of food intake during the refeeding period. (**C**) Total, day and night voluntary exercise in wheel at the end of the refeeding period; FRW mice showed an alteration in the daily distribution of their physical activity. (**D**,**E**) Acyl- (**D**) and desacyl-ghrelin (**E**) after one day of refeeding (D51). (**F**,**G**) Acyl- (**F**) and desacyl-ghrelin (**G**) after 20 days of refeeding (D70). Data are expressed as mean ± SEM; *n* = 6/group. Dotted lines in **B** represent the percentage value corresponding to the food eaten by ALW mice. * *p* < 0.05; ** *p* < 0.01. ALW: ad libitum and wheel; FRW: food restriction and wheel.

Fifty days after the beginning of quantitative food restriction for the FRW group, ad libitum food regimen was fully restored. The body weight of the FRW mice was rapidly restored after one day of refeeding (Figure 3A). In 4 days, FRW mice exhibited higher food intake as compared to the ALW group (D1: 213% of ALW food intake, D2: 167%, D3: 147%, Figure 3B).

It was not possible to properly measure physical activity during early refeeding from D51, because of the high binge-eating-like behaviour that interfered with FRW mice daily exercise. However, at D70, when FRW mice body weight was completely restored, activity in running wheels was significantly lower as compared to ALW mice during night-time only (*U* = 0, *p* = 0.007, Figure 3C).

On the first day of the refeeding period (D51), AG and DAG plasma concentrations were significantly decreased in FRW mice compared to ALW mice (respectively *U* = 3, *p* = 0.015; *U* = 3, *p* = 0.015, Figure 3D,E), whereas at D70, no difference was noted between the two groups for AG (*U* = 13, *p* = 0.792, Figure 3F) and DAG (*U* = 10, *p* = 0.429, Figure 3G).

At D70, night and total activities in ALW mice were positively correlated to AG (respectively, rho = 0.887, *p* = 0.018; rho = 0.888, *p* = 0.018), but not DAG. In contrast, total activity in FRW mice was positively correlated to DAG (rho = 0.96, *p* = 0.01) with a tendency only for day activity (rho = 0.820, *p* = 0.089) but not AG (Table 3).

## 4. Discussion

In the present study, we aimed to improve our understanding of the link between plasma concentrations of the two isoforms of ghrelin and the level of physical activity in condition of chronic food restriction and during nutritional recovery. In AN patients, under an inpatient therapeutic program, we observed that one month after discharge only plasma DAG concentrations were negatively correlated to BMI and positively to the level of physical activity. In keeping with this observation in AN patients, we also showed in mice that after two weeks of nutritional recovery, plasma DAG concentrations were positively correlated with diurnal physical activity.

In AN, refeeding is accompanied by a significant reduction in plasma ghrelin concentrations [23,48]. To our knowledge, only one study reported a differential evolution of plasma AG and DAG concentrations during nutritional recovery in five women with restrictive-type AN [49]. Plasma DAG concentrations decreased more rapidly than AG in the early stage of hospitalisation and after 8 weeks they remained significantly lower than in ten control subjects [49].

Relapse after hospitalisation is a major clinical challenge, especially within the first year following treatment [44,50]. Thus, the identification of factors influencing recovery is a research priority for AN [51]. During hospitalisation, the therapeutic program followed by our AN patients involves reduction of exercise and physical activity limitations and both quantitative and qualitative food intake modifications. During this period, we observed that plasma concentrations of total ghrelin, AG and DAG decreased rapidly, as previously demonstrated in longitudinal studies [23,49]. The therapeutic program includes a progressive exposure to home after weight recovery and before discharge, with regular home visits. However, the period following weight recovery remains a challenge both for patients and caregivers, most patients losing weight one month after discharge and resuming their physical hyperactivity routine. In the present study plasma AG concentrations increase faster than DAG after return in an ecological environment. More specifically, BMI and DAG are negatively correlated. Recent data showed that physical activity correlated positively with total plasma ghrelin levels in acute state of AN [37]. Here, only DAG plasma concentrations at discharge were associated with increased physical activity one month later. Although the follow-up was too short to conclude about a possible relapse, our data further support that early variations of body weight and physical activity may be influenced by metabolic factors at discharge. Length of hospitalisation and balance between inpatient and intensive outpatient treatment might benefit from objective metabolic biomarkers, such as AG and DAG.

To better decipher the potential interaction between daily physical activity and metabolic alterations, we used a preclinical mouse model of chronic food restriction associated with voluntary physical activity, in which metabolic parameters have been characterised previously following either a short- (2 weeks) or long-term (8 weeks) protocol [45,46]. In the present study, AG and DAG plasma concentrations were assessed at different key stages of the protocol, using selective and sensitive immunoassays [29]. We first assessed such variations in mice submitted to a two-week food restriction that had the ability to run in a wheel. The association between ghrelin and exercise had previously been demonstrated in the “activity-based anorexia” (ABA) model. Indeed during 5 days of ABA protocol, GHRS-R1a antagonism inhibited food anticipatory activity (FAA) in mice [38] and the motivational drive to eat in rats [52]. Moreover, ghrelin knockout mice exhibited a lower FAA in wheel during a time-restricted feeding protocol and acute administration of GHRP-6, a GHSR-1a agonist, was sufficient to enhance the amount of voluntary exercise in ghrelin KO mice [53]. This increase of FAA induced by GHRP-6 is mediated by an increase of dopaminergic activity in the nucleus accumbens [53]. These data are of importance since they emphasise how ghrelin, usually studied for its involvement in the modulation of food intake [54], is essential to initiate voluntary exercise in parallel to feeding behaviour. Here, in our quantitative food-restricted model, we consolidated the data obtained by Mifune et al. in their time-food restricted model [53]. Indeed, only the food-restricted mice that developed the highest FAA displayed the highest plasma concentrations of both AG and DAG. The mechanisms by which animals anticipate feeding remain yet unresolved (see. Mistlberger (1994) for review) [55]. Food anticipatory activity might be induced by both food-inducible oscillators—the precise location into the brain of which remains to be determined [56,57]—and circadian-clocks located in the suprachiasmatic nucleus [58]. LeSauter et al. (2009) suggested that stomach-producing ghrelin cells contain food-entrainable oscillators [59]. They showed that intraperitoneal ghrelin administration in non-deprived mice, but in the absence of food, induced an increase in the locomotor activity while mice lacking ghrelin receptors displayed a significant reduction of FAA. Acylated ghrelin appears to stimulate both the appetitive (anticipatory locomotor behaviour) and the consummatory component (food intake) of feeding behaviour. However, AG does not appear to be necessary for FAA although anticipatory activity rhythms may exhibit a reduced peak level or duration in *ghsr* −/− mice; supporting a modulatory influence of ghrelin rather than a full food-inducible oscillator role [56,60,61,62,63]. Although the potential role of DAG as a hormone remains a matter of debate, DAG is suggested to be a signalling molecule that has specific targets, including the brain, with mostly opposite and independent effects to AG on food intake and glucose homeostasis [27,30,64,65]. To our knowledge, a relation between DAG and physical activity anticipatory to feeding had not been previously demonstrated in food-restricted rodents. An elegant study on bird migratory behaviours demonstrated that injections of DAG decrease food intake and increase migratory restlessness [66]. Indeed, ethologic condition of migration associates voluntary exercise to voluntary immediate food renunciation permitting long-term gain for the species [67]. As mentioned by Guisinger et al. (2003) “AN’s distinctive symptoms of restricting food, denial of starvation, and hyperactivity are likely to be evolved adaptive mechanisms that facilitated ancestral nomadic foragers leaving depleted environments; genetically susceptible individuals who lose too much weight may trigger these archaic adaptations.” [67]. Although highly speculative and lacking enough empirical substantiation, these findings prompted us to determine whether the evolution of AG and DAG in relation with physical activity during and after feeding evolved similarly to establish them as reliable post-remission predictors.

The food restriction protocol was thus extended to 50 days followed by a long-term nutritional recovery protocol. Such a protocol better mimicked the physiological changes that occur during weight recovery in AN patients. In this perspective, our preclinical model may fulfil face validity (phenomenological similarities for the physiological alterations) and predictive validity, criteria that are essential to model pathology [68]. After long-term food restriction, plasma AG and DAG concentrations remained elevated. Then, the prolongation of the protocol impacted the day-night exercise setting out even after 20 days of nutritional recovery, despite the technical necessity of maintaining two mice per cage to limit social stress. Nevertheless, this suggested that after 20 days of nutritional recovery, the voluntary physical activity remained differentially correlated with AG or DAG, in control or food restricted mice respectively. Indeed, DAG positively correlated with exercise performed during the day, only in the FRW group.

The effects of exercise on plasma total or AG plasma ghrelin levels have been investigated in multiple human and rodent studies although the results have been inconsistent, demonstrating either a decrease, increase, or no change [40,69]. Only total and AG concentrations have been measured. To our knowledge, no study described the impact of physical activity on DAG. Here, we benefited from the development of selective and sensitive assays, validated both in humans (personal data) and rodents [29]. Our results converge in both rodents and humans and highlight the importance of the balance between AG and DAG. Indeed on one side, AG is rapidly converted into a DAG because of the rapid action of blood esterases [70]. On the other side, the ghrelin *O*-acyl transferase (GOAT) that permits the octanoylation of ghrelin (AG) is now considered to be a key regulator in energy metabolism and hedonic feeding [71,72,73]. DAG has long been considered to be an inactive product of degradation of AG but subsequent data suggest that it is a metabolically active peptide acting through a yet unknown receptor [27]. Mostly, DAG antagonises but sometimes acts synergistically with AG, since it can bind and activate the AG receptor but with a lower affinity in vitro and in vivo [74]. However, DAG does not reach the necessary concentrations in tissues to do so, at least under physiological conditions [27]. Overall, studies in rodents and humans support a role of DAG to decrease body weight, food intake and body fat [75,76]. Indeed, DAG overexpressing mice exhibit a decrease in body weight, food intake, fat pad mass weight accompanied by a modest decrease in linear growth [76]. These physiological changes are attributable to (1) the decrease in gastric emptying and (2) an anorexigenic effect of DAG mediated by a specific activation of hypothalamic neurons [76,77]. In the present FRW model, intraperitoneal injection of DAG increased physical activity [78]. Therefore, we hypothesise an indirect action of DAG on physical activity. Indeed, GOAT activity has been detected in the hypothalamus and pituitary and its hypothalamic expression is nutritionally regulated [73,79]. Thus, increased DAG plasma concentrations during chronic food restriction might indirectly activate GHS-R via a local hypothalamic conversion into AG, leading to an adaptive increase of physical activity [80].

The present clinical data enlightened the potential role of DAG in recovery processes in AN and suggested a potential negative impact of DAG on weight recovery. Such interpretation might be paralleled with data obtained from the mouse model where increased plasma DAG correlated with an unusual physical activity during daytime, since rodents are used to exercise during night-time. This may reflect a sustainable alteration of physiological regulation of feeding-relative behaviours away from a long- period of caloric restriction that can interfere with a proper recovery. DAG has also been implicated in myogenesis and thus may protect the muscle integrity along weight loss [54,81]. Finally, we cannot exclude the role of elevated DAG plasma concentrations in the development of osteopenia and osteoporosis even if DAG effects on bone physiology are currently very limited and contradictory. On one side, DAG stimulates human osteoblasts proliferation in the absence of GHS-R1a [82]. On the other side, mice overexpressing DAG show a moderate decrease in their linear growth suggesting an impairment of the skeletal integrity [76]. Further mechanistic studies will help to decipher how AG and DAG modulate both muscle and bone integrity and functioning.

Translational data might be a prerequisite to stress the mechanisms that support a successful recovery in AN. On one hand, ghrelin agonists induced motivation to exercise through activation of the central reward circuit [53]. Furthermore, central administration of ghrelin enhances exercise through dopamine release in the nucleus accumbens [83]. In AN, an alteration of the reward circuit is now well accepted [11]. Further studies are needed to determine the action of DAG on this brain circuit. On the other hand, several data have linked the body temperature with the level of activity both in mice, in rats and in AN patients [84,85]. Thus, increasing physical activity through a direct or indirect action of AG or DAG could maintain appropriate body temperature in AN.

*Limitations and perspectives.* There are several limitations to this study. First, the small size of the clinical sample prevents generalisation and calls for replication. Second, we need to be cautious with the interpretation of data related to physical activity measurement in clinical and preclinical experiments. In the clinical sample, we used an internationally valid self-report assessment (IPAQ). Actimetry, heart rate monitoring, and recent progress on portable devices would allow investigating physical activity based on objective measures, which would be more accurate. Indeed, physical activity in AN patients is underestimated by subjective assessment (IPAQ) when compared with objective (Actiheart) measurement [86]. We also confirmed that objective assessment of physical activity could be more informative than subjective ratings, to reflect for example cognitive specificities of anorexia nervosa [87]. As surprising as it may seem, physical activity assessment in AN is so far not well defined, and there is currently no real consensus on how to measure it. In future research, it seems necessary to assess the different aspects of physical exercise conjointly (obligatory exercise, addiction exercise, commitment for exercise, reasons for exercise, isometry…) [88]. Recent studies support the need to investigate more in depth this unsuitable behaviour in a condition of reduced energy supplies [2,89]. Third, the choice to maintain as long as possible two restricted mice in a cage equipped with a wheel to avoid social stress and hypothermia, could limit the measurement of physical activity. However, separate experiments done in metabolic cages (where the ALW and FRW mice were singly housed for 5 to 6 days), validated that singly housed FRW mice exhibited similar FAA after 15 days of protocol (Duriez et al., unpublished data, [45]). For the welfare of the animals and to avoid stress isolation and hypothermia induced by a long-term caloric restriction, mice were maintained as two per cage. The recording of physical activity reflected the activity of two mice that usually run two by two in the wheel (ALW or FRW, personal observation). We also observed that the nutritional recovery was extremely rapid in our animal experiment, with a rapid body weight gain, that is consistent with a faster metabolism observed in rodents than in human. It might be interesting to validate another protocol of slow nutritional refeeding monitored by the investigator. Altogether, this reflects the limitations of animal modelling of psychiatric disorders. The rapid decrease of AG and DAG plasma concentrations, rapid weight gain and binge-eating like behaviour in the first three days of nutritional recovery suggest the persistence of food directed motivation after a long caloric restriction period. However, our conclusion asks the question to compare progressive to rapid weight recovery in mice. Indeed, clinical practice reported in some cases a rapid weight recovery in AN, especially in the case of clinical switch from AN to bulimia nervosa [90,91]. Ghrelin gene variants may also predict crossover rate from restricting-type AN to binge-purging subtype or bulimia nervosa [92]. We need to know whether the changes in AG and DAG during a rapid or a progressive weight recovery associated or not with binge-eating crisis forecast sustainable altered eating behaviour. Finally, weight gain cannot be considered as the unique remission factor. Preclinical models appear then to be crucial tools to decipher long-term metabolic alterations after full weight recovery and despite their limitations, they could permit to test pharmacological options. Shall DAG-signalling be a pharmacological target in AN?

## Figures and Tables

**Table 1:** Demographic, anthropometric and socio-economic characteristics of anorexia nervosa in the clinical samples. Data are presented as mean +/− SD. T0: at admission; T1: 50–70% of target BMI reached; T2-T3: weight recovered patients with evaluation one month post discharge; AN: anorexia nervosa; AN-R: anorexia nervosa restrictive type; AN-BP: anorexia nervosa binge-eating/purging type; BMI: body mass index; EDI2: Eating Disorder Inventory version 2.

	T0 (*n* = 29)		T1 (*n* = 28)		T2-T3 (*n* = 13)	
	Mean +/−SD	%	Mean +/−SD	%	%	%
**Age (years)**	25.8+/−6.8		26.2 +/−6.44		26+/−7	
**AN-Duration (years)**	7.4+/−5.4		7.4+/−5.5		8.8+/−6.5	
**Age of onset**	18.4+/−3.8		18.4 +/−3.9		17.2+/−1.9	
**AN Subtype**						
AN-R		59%		57%		60%
AN-BP		41%		43%		40%
**BMI at inclusion (kg/m^2^)**	14.6+/−1.3		14.6+/−1.4		14.8+/−1.2	
**Partner ship**						
Single		79%		78%		69%
In a relationship		21%		22%		31%
Daily psychiatric drugs		62%		62%		62%
**EDI-2 at inclusion**						
Total	116+/−5		118+/−52		114+/−53.7	
Drive for thinness	11.9+/−7.4		12.2+/−7.3		11.5+/−8.6	
Bulimia	4.9+/−7		5.1+/−7.1		5+/−7.62	
Body dissatisfaction	17+/−6.9		17.1+/−7		17+/−6.7	
Ineffectiveness	16+/−8.9		16.5+/−8.7		15.5+/−9.3	
Perfectionism	7.5+/−4.4		7.3+/−4.4		7.7+/−4.6	
Interpersonal distrust	9.4+/−4.8		9.3+/−4.9		9.4+/−4.5	
Interoceptive awareness	14.7+/−7.3		14.8+/−7.4		14.2+/−5.9	
Maturity fears	8.3+/−6.5		8.6+/−6.4		8.2+/−7.5	
Asceticism	9+/−5.74		9.3+/−5.8		9+/−6.32	
Impulse Regulation	7+/−7.25		7+/−7.3		6+/−7.4	
Social Insecurity	11+/−5.52		10.8+/−5.4		11+/−6.3	

**Table 2:** Correlations between BMI, physical activity, AG and DAG plasma concentrations in the clinical samples after weight restoration and one-month post discharge.

	BMI T2	PA T2 ^1^	AG T2	DAG T2	BMI T3	PA T3	AG T3	DAG T3^1^	Δ BMI	Δ PA	Δ AG	Δ DAG
**BMI T2**	—											
**PA T2 ^1^**	0.007	—										
**AG T2**	0.107	−0.322	—									
**DAG T2**	−0.378	−0.609 *	0.205	—								
**BMI T3**	0.533	0.111	−0.264	−0.607 *	—							
**PA T3**	−0.188	0.165	0.159	0.192	−0.038	—						
**AG T3**	−0.107	0.146	0.369	0.186	−0.510	0.527	—					
**DAG T3 ^1^**	−0.302	0.069	0.346	0.319	−0.494	0.301	0.445	—				
**Δ BMI**	0.087	0.141	−0.369	−0.511	0.890 ***	0.050	−0.543	−0.278	—			
**Δ PA**	−0.121	−0.664 *	0.468	0.587 *	−0.204	0.613 *	0.412	0.159	−0.175	—		
**Δ AG**	−0.190	0.085	−0.492	0.002	−0.257	0.344	0.628 *	0.143	−0.200	−0.006	—	
**Δ DAG**	−0.222	0.198	0.355	−0.063	−0.101	−0.015	−0.072	0.791 **	0.001	−0.113	−0.364	—

T2: after weight restoration (90–100% BMI target); T3: one-month post discharge. BMI: body mass index; PA: physical activity measured by IPAQ; AG: acyl-ghrelin; DAG: desacyl-ghrelin. ^1^ Shapiro-Wilk *p* < 0.05: Spearman’s rank correlation coefficient is indicated; Δ: delta between T2 and T3. * *p*<0.05; ** *p*<0.01; *** *p*<0.001.

**Table 3:** Correlation between physical activity in wheel (total, day and night) and acyl- and desacyl-ghrelin plasma concentrations, in FRW and ALW mice at D70 (20 days of refeeding). Spearman’s rank correlation coefficient is indicated by rho.

		ALW		FRW	
		AG	DAG	AG	DAG
**Activity in Wheel**	*Total*	0.887 *	0.609	0.699	0.960 *
	*Day*	−0.297	−0.407	0.569	0.820
	*Night*	0.888 *	0.614	0.117	0.049

* *p* < 0.05. Significant values are indicated in bold. AG: acyl-ghrelin; DAG: desacyl-ghrelin; ALW: ad libitum and wheel; FRW: food restriction and wheel.
